# Peripheral Nerve Blocks for Hip Fractures

**DOI:** 10.3390/jcm13123457

**Published:** 2024-06-13

**Authors:** Iyabo O. Muse, Brittany Deiling, Leon Grinman, Michael M. Hadeed, Nabil Elkassabany

**Affiliations:** 1 Department of Anesthesiology, University of Virginia Health System, Charlottesville, VA 22908, USA; bd8hp@uvahealth.org (B.D.); hzg6vu@uvahealth.org (L.G.); ekq8wm@uvahealth.org (N.E.); 2 Department of Orthopedic Surgery, University of Virginia Health System, Charlottesville, VA 22903, USA; mmh2j@uvahealth.org

**Keywords:** fascia iliaca block, pericapsular nerve group (PENG) block, lateral femoral cutaneous nerve

## Abstract

The incidence of hip fractures has continued to increase as life expectancy increases. Hip fracture is one of the leading causes of increased morbidity and mortality in the geriatric population. Early surgical treatment (<48 h) is often recommended to reduce morbidity/mortality. In addition, adequate pain management is crucial to optimize functional recovery and early mobilization. Pain management often consists of multimodal therapy which includes non-opioids, opioids, and regional anesthesia techniques. In this review, we describe the anatomical innervation of the hip joint and summarize the commonly used peripheral nerve blocks to provide pain relief for hip fractures. We also outline literature evidence that shows each block’s efficacy in providing adequate pain relief. The recent discovery of a nerve block that may provide adequate sensory blockade of the posterior capsule of the hip is also described. Finally, we report a surgeon’s perspective on nerve blocks for hip fractures.

## 1. Introduction

Hip fracture is common among the elderly population (age > 65 yrs.) and is one of the leading causes of increased morbidity and mortality in the geriatric population. Surgical treatment within 48 h is often recommended to decrease perioperative morbidity [1]. There are several surgical procedures indicated based on the specific type of fracture and patient characteristics including open reduction internal fixation, hip hemiarthroplasty, and/or total hip arthroplasty. For optimal recovery, decreased postoperative delirium, and the early mobilization of patients, an effective pain management protocol is necessary. Pain management often consists of multimodal therapy which includes non-opioids, opioids, and regional anesthesia techniques. Peripheral nerve blocks, a component of regional anesthesia, have been shown to improve postoperative pain, reduce morphine consumption, and increase patient satisfaction after hip surgery [2]. Studies show that the use of peripheral nerve blocks and multimodal analgesia techniques may reduce the incidence of delirium and facilitate early mobilization, thus ultimately reducing morbidity and post-op 1-year mortality after hip surgery [3,4]. In this article, peripheral nerve blocks used to provide analgesia for hip fractures and their efficacy are discussed.

## 2. Anatomic Innervation of Hip Joint

**Figure 1:**
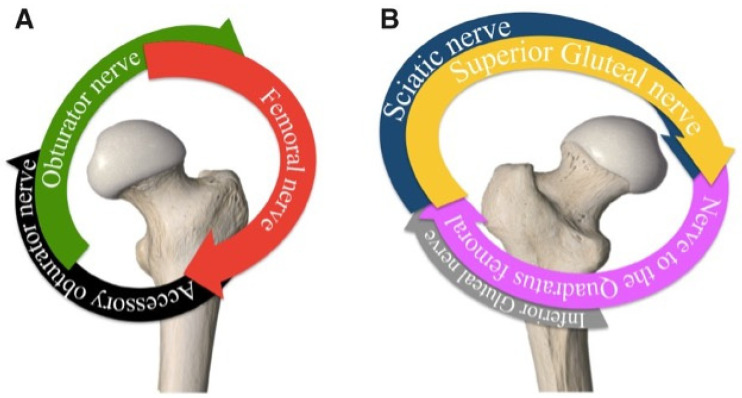
Schematic diagram of the nerve supply of quadrants of the hip joint: (**A**) anterior and (**B**) posterior views. Reproduced with permission from Laumonerie et al., Pain Medicine; published by Oxford University Press, 2021 [6].

**Figure 2:**
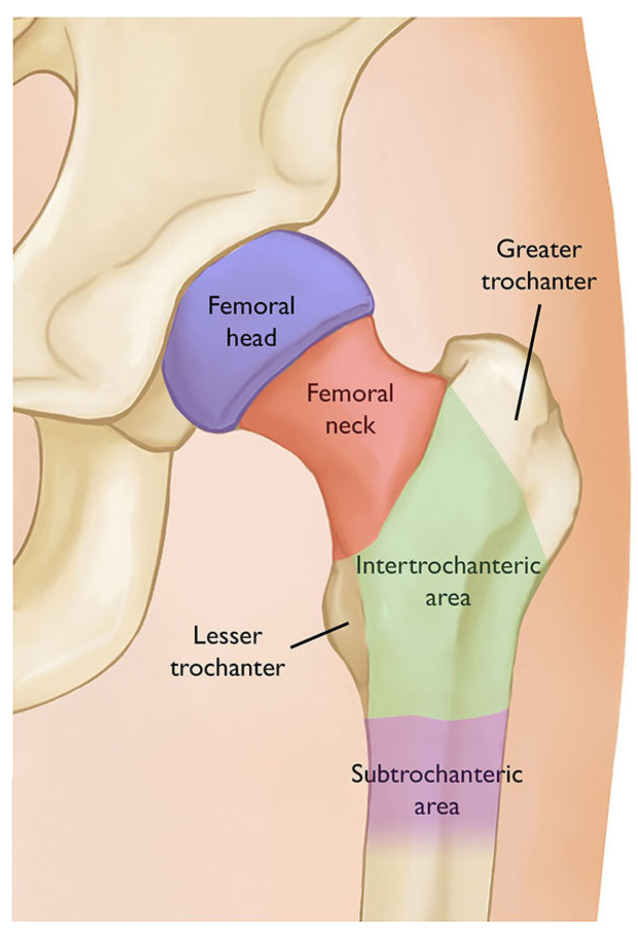
The areas of the femur. Most hip fractures occur in the femoral neck or intertrochanteric area. Reproduced with permission from Fischer, S and Gray, J, OrthoInfo; published by American Academy of Orthopedic Surgeons, 2020 [7].

The target nerves of the hip joint arise from the lumbar plexus (L1–L4), the lumbosacral trunk of the sacral plexus (L4–L5), and the sacral spinal nerves (S1–S4). The femoral nerve, obturator nerve, and the accessory obturator nerve supply the anterior capsule of the hip; the sciatic nerve and the nerve to the quadratus femoris mostly supply the articular branches to the posterior capsule of the hip joint. It is understood that the anterior capsule supplied by the femoral nerve, accessory obturator nerve, and the superior labrum are the predominant causes of pain in the hip joint [5,6] (Figure 1A,B). However, the site of pain is determined by the area of the femur where the fracture occurred. Most hip fractures occur in the femoral neck or intertrochanteric area (Figure 2) [7]. 

## 3. Peripheral Nerve Blocks

There are several peripheral nerve blocks that can provide analgesia to the hip joint. These nerve blocks include the fascia iliaca block (FICB, supra-inguinal/infra-inguinal), femoral nerve block, lateral femoral cutaneous nerve block (LFCN), Pericapsular Nerve Group (PENG) block, quadratus lumborum block (transmuscular–anterior), lumbar plexus nerve block, sciatic nerve block, and the newly explained posterior pericapsular deep-gluteal (PPD) block. 

### 3.1. Fascia Iliaca Block (FICB)

The fascia iliaca block (FICB) is an injection of local anesthetic into the fascia iliaca compartment and targets the femoral nerve (FN), obturator (OBN), and lateral femoral cutaneous nerve (LFCN) between the fascia iliaca and underlying iliacus muscle [8]. However, it is important to note that the FN and the LFCN is more reliably blocked by FICB, while the OBN block requires a larger volume to be blocked. Traditionally, the fascia iliaca block is performed inferior to the inguinal ligament using a landmark-based technique [9]. This technique involves drawing a line between the pubic tubercle to the anterior superior iliac spine (ASIS) and dividing this line into three sections spaced equally apart. The point of entry of the block needle is 1 cm caudal to the point dividing the lateral third and medial two-thirds of the line. The needle is then inserted perpendicular until two loss of resistances are appreciated, the first being the fascia lata and the second loss of resistance being the fascia iliaca. After a negative aspiration, local anesthesia is injected into this space under the fascia iliaca [10]. 

**Figure 3:**
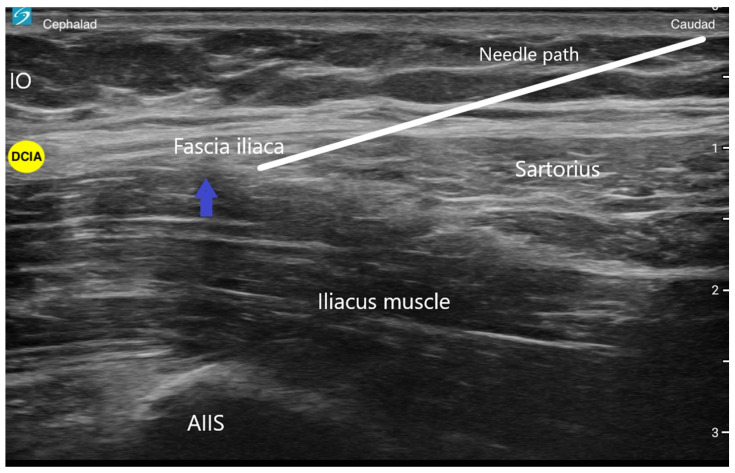
This is an ultrasound image of a supra-inguinal fascia iliaca (SIFI) block. The white line represents the direction of the needle. The blue arrow represents the location of local anesthetic injection. IO (internal oblique muscle); AIIS (anterior inferior iliac spine); DCIA (deep circumflex iliac artery).

A double-blind randomized control trial demonstrated superior pain relief, less opioid consumption, and less opioid-related sedation when the conventional infra-inguinal fascia iliaca block was compared to standardized systemic morphine analgesia for the management of acute hip fracture pain [10]. A systematic review found the loss-of-resistance-technique fascia iliaca compartment block to be an effective and safe tool for preoperative pain management in patients with hip fractures [11]. With the movement away from landmark-based techniques, an ultrasound-guided technique of the FICB, called a supra-inguinal fascia iliaca block (SIFI), was described and demonstrated to be a more reliable block of the target nerves [12]. This novel approach was created by Hebbard et al. with cadaveric models that showed extensive injectate spread throughout the iliac fossa when using an ultrasound-guided supra-inguinal approach [13]. They describe using a high-frequency probe placed parasagittally over the inguinal ligament close to the ASIS with an in-plane technique and needle entry point 2–4 cm below the inguinal ligament. The needle is advanced through the fascia iliaca at the level of the inguinal ligament, and through hydrodissection, local anesthetic is deposited between the iliacus muscle and the fascia iliaca and superiorly into the iliac fossa, using approximately 30–40 mL (Figure 3) [13]. An important landmark for SIFI is the deep circumflex iliac artery which lies laterally on top of the fascia iliaca. The artery serves as a landmark to confirm adequate local anesthetic spread between the fascia planes. A randomized control trial concluded that for total hip arthroplasty, the supra-inguinal fascia iliaca block provides superior analgesia in the first six hours postoperatively and significantly less morphine consumption in the first 24 h compared to the infra-inguinal approach [14]. Specifically for hip fractures, a retrospective study found the SIFIB group had statistically significantly lower postoperative 24 h opioid consumption compared to the control group, and thus reduced opioid-related respiratory depression after hip fracture surgery in older-old patients (over 80 years old) [15]. 

The fascia iliaca block provides good analgesia for the hip joint and improves patient satisfaction, regardless of which approach is used. A retrospective study showed a significantly shorter length of stay and lower pain scores on postoperative days 2 and 3 in hip fracture patients who had an ultrasound-guided SIFI catheter compared to the no-block group [16]. However, a possible complication to FICB is quadricep weakness from the spread of local anesthetic to the femoral nerve. Thus, caution should be taken if ambulation is wanted on the day of surgery. 

### 3.2. Femoral Nerve Block

**Figure 4:**
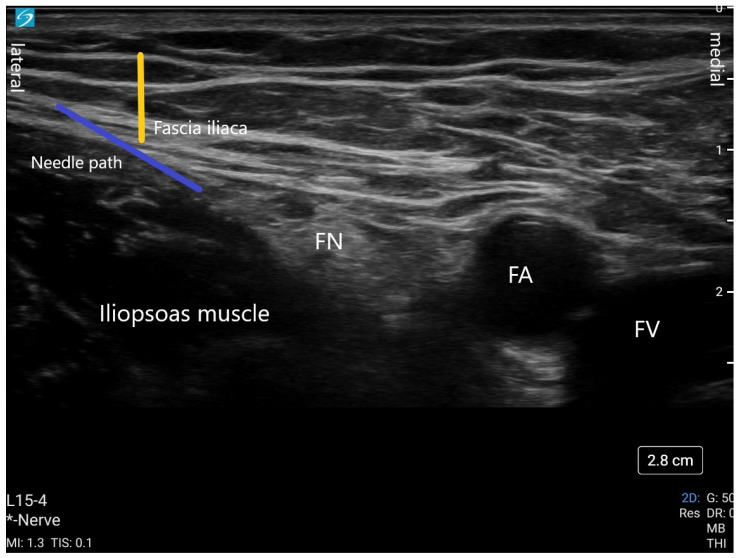
This is an ultrasound image of a femoral nerve block. The yellow line indicates the fascia iliaca plane. The blue line represents the needle trajectory. Local anesthetic is deposited below the nerve and above the nerve. FN (femoral nerve); FA (femoral artery); FV (femoral vein); * Nerve (ultrasound setting is in Nerve mode).

The femoral nerve block (FNB) continues to be one of the mainstays of regional techniques for pain management after hip fracture. Its ease of performance lends itself to accessible mastery and applicability. The femoral nerve supplies sensory fibers to the hip joint in addition to motor and sensory supply to the thigh. It arises from lumbar spinal roots L2-L4. The nerve is commonly blocked posterior to the inguinal ligament where it lies lateral to the femoral artery. An FNB is often conducted with ultrasound guidance using a high-frequency linear probe at the femoral crease with the patient lying supine. A lateral approach using an in-plane needle technique is used to inject local anesthetic (15–20 mL) either above or below the nerve (Figure 4). A continuous nerve catheter can also be placed using ultrasound guidance for prolonged analgesia. 

There have been several studies in the past five years relating to the femoral nerve block’s pain-modulating effectiveness, its tendency to decrease VAS pain scores, its effect on early post-op mobility, and low evidence of reducing delirium in a highly susceptible population [5,17,18,19]. More recent randomized controlled trials (RCTs) continue to show a benefit in terms of pain control. An RCT of 110 patients in Turkey showed that when comparing patients with femoral blocks to patients just receiving acetaminophen IV, the patients with FNBs had lower VAS pain scores during positioning for intra-op spinal anesthesia [18]. A study of 407 elderly patients in Switzerland with hip fractures showed a significant reduction in the amount of post-op opioid usage as compared to those that did not receive blocks [19]. 

In all, the FNB continues to be a powerhouse of the regional world in terms of regional techniques for hip fracture patients. Some of our strongest evidence, the Cochrane review by Guay et al., leans heavily on the FNB, though it includes data of patients being administered other types of blocks [5]. As ultrasound techniques continue to improve, the literature will start to reflect whether there is even more benefit being gleaned than is currently reported.

### 3.3. Lateral Femoral Cutaneous Nerve (LFCN) Block

**Figure 5:**
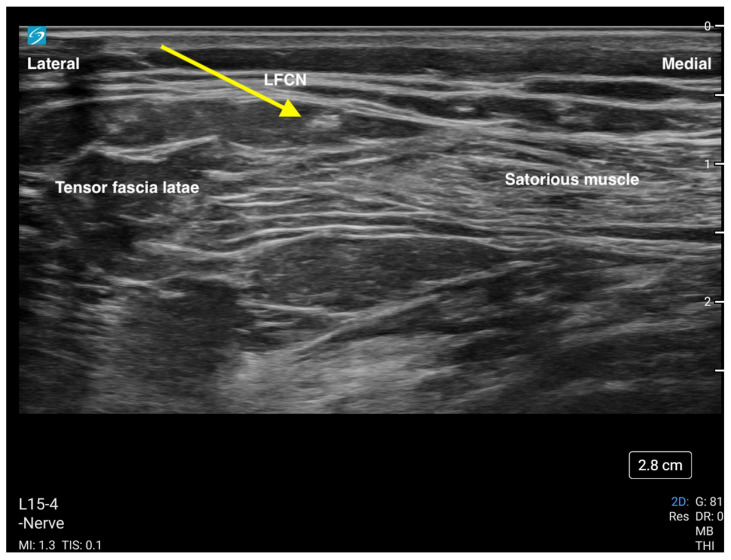
This is an ultrasound image of the lateral femoral cutaneous nerve (LFCN). The yellow arrow is pointing to the nerve. The nerve is blocked with a needle in the direction from lateral to medial.

The lateral femoral cutaneous nerve (LFCN) block is a useful adjunct to the other nerves (femoral nerve, FICB) commonly blocked for hip fracture surgery. Its distribution at the lateral side of the leg allows for easier blockade in the context of a lateral surgical incision. The LFCN is indicated for analgesia for surgery of the anterolateral thigh, i.e., open reduction internal fixation of the hip, hip arthroplasty and/or hemiarthroplasty, muscle biopsy, and to treat meralgia paresthetica. The block is performed using a high-frequency linear ultrasound probe placed in a transverse position just distal to the anterior superior iliac spine (ASIS). The nerve can be identified as a hyperechoic structure superficially and between the sartorius and tensor fascia latae muscles (Figure 5). Depositing 3–5 mL of local anesthetic by inserting a needle using an in-plane approach and deep to the fascia lata should provide good analgesic coverage of the lateral thigh. 

Most recent studies of the LFCN occur in the context of another block such as the PENG block or the fascia iliaca block. In that context, it has been shown to be beneficial toward overall pain control and opioid sparing effect. One RCT from South Korea in 2024 placed patients into two groups, one receiving local infiltration by the surgeon (LIA) and the other with LIA + PENG and LFCN. This study showed significant reductions in pain scores and opioid consumption [20]. Another study combining PENG and LFCN evaluated whether this combination could be used as a primary block in patients. They compared three groups of patients (120 total) undergoing hip fracture surgery: GA, spinal, and RA. They found numerous benefits in the regional-only group and they concluded that PENG and LFCN may decrease postoperative pain and the length of hospital stay and allow for greater ambulation in the early postoperative period for patients with hip fractures [21]. The LFCN block was again studied as a part of a primary anesthetic, this time with the FNB as the other half, in hip fracture patients with a high co-morbidity load. The authors found the combination of blocks to be sufficient to get the patient through intramedullary nail placement as a salvage anesthetic when spinal or GA is not appropriate [22]. 

As the above literature shows, the LFCN block continues to serve as an important supplemental block for patients experiencing hip fracture pain. Though not utilized in isolation, its anatomic distribution makes it ideal as a rescue block for patients who have had lateral/ posterolateral hip incisions, especially in combination with blocks that are known to affect the anterior capsule. 

### 3.4. Pericapsular Nerve Group (PENG) Block

**Figure 6:**
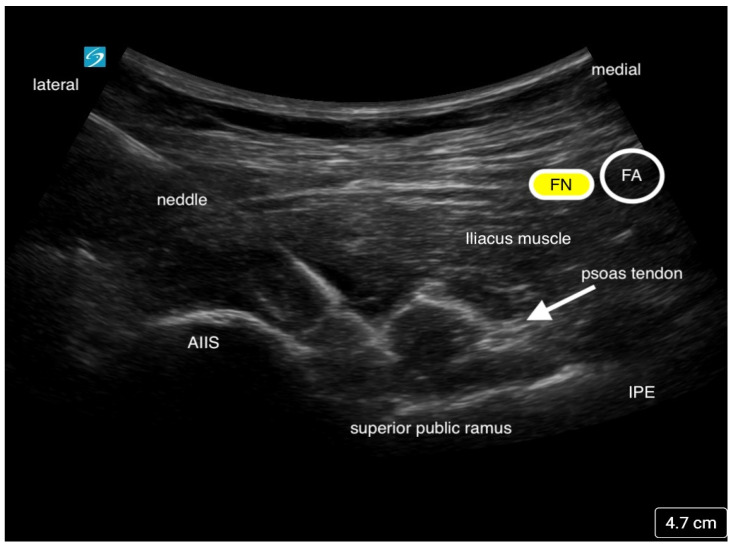
This is an ultrasound image of the Pericapsular Nerve Group Block (PENG). Local anesthetic is injected below the psoas tendon. The needle approach is from lateral to medial. The femoral nerve (FN) and femoral artery (FA) is seen medially.

The Pericapsular Nerve Group (PENG) block is a fascial plane block that was developed by Giron-Arango et al. at Toronto Western Hospital and the University of Toronto to block the high articular branches of the femoral (FN), obturator (ON), and accessory obturator nerves (AON) to the hip joint [23]. These branches are mostly responsible for the nociceptive pain in the anterior and superolateral capsule of the hip joint. The FN and AON are often found between the anterior inferior iliac spine (AIIS) and the iliopubic eminence (IPE), thus making it easier to target both nerves. The block is performed with the patient in the supine position. Local anesthetic is deposited using a curvilinear low-frequency ultrasound probe with an in-plane technique from lateral to medial. The needle tip is placed in the fascial plane between the psoas tendon and the ilium (Figure 6) [23,24]. As described by Giron-Arango and colleagues, an optimal ultrasound image should include the AIIS, IPE, psoas tendon, iliopsoas muscle, and more superficially, the femoral neurovascular bundle [23]. Typically, 10–20 mL of local anesthetic is adequate to provide effective analgesia if there is fluid spread along the plane displacing the psoas muscle tendon.

The utilization of the PENG block in various studies has shown its superior analgesic effectiveness in lowering opioid consumption, decreasing the incidence of motor blockade, and shortening discharge time as compared to patients with no nerve block, a femoral nerve block, and an infra-inguinal fascia iliaca block with hip fractures [23,25,26,27]. In a single-center, double-blind, randomized controlled trial performed by Lin, Xufeng et al. in Singapore General Hospital, they found that the PENG block was superior to a sham block at reducing acute traumatic pain at 30 min (*p* < 0.01) and 3 h post block (*p* < 0.05) and reducing opioid consumption (*p* < 0.05) following hip fracture [28]. Although a PENG block is good at providing analgesia, it cannot be used as the sole anesthetic for hip surgery. It is often combined with LFCN, FICB, or FNB to provide more comprehensive analgesia for hip surgery. 

### 3.5. Lumbar Plexus Nerve Block

The sensory innervation of the hip involves branches of both the lumbar and sacral plexus. The lumbar plexus is composed of L1–L4 nerve roots, which enter the psoas major muscle after they exit the intervertebral foramina [29]. These lumbar roots terminate as the femoral, lateral femoral cutaneous, obturator, genitofemoral, iliohypogastric, and ilioinguinal nerves as they pass through the psoas major muscle. Cadaveric studies have concluded that the lumbar plexus lies within the psoas major muscle, but the location of the obturator nerve within the psoas major muscle may vary [30]. 

The lumbar plexus block, also known as the psoas compartment block, targets the fascial plane within the posterior aspect of the psoas major muscle. The anterior approach to the lumbar plexus block is known as the three-in-one block (it blocks the femoral, obturator, and LFCN nerves), and many different approaches to the posterior approach have been described [31]. Winnie et al. first described the posterior approach to the lumbar plexus block, but the inadvertent neuraxial spread causing bilateral anesthesia has led to concerns with its usage [32,33]. The lumbar plexus can be blocked within the psoas major muscle using different techniques including paraesthesia, loss of resistance, nerve stimulation to elicit quadricep muscle contraction, and, in more recent years, ultrasound guidance. The adequate volume of local anesthetic is between 20 and 25 mL [34].

In addition to its analgesic benefits in hip fracture patients, the lumbar plexus block can also be used as the primary anesthetic. In comparing a single-shot lumbar plexus block as the primary anesthetic to a subarachnoid block, one study showed the lumbar plexus blockade to provide more stable intraoperative hemodynamics and a longer duration of postoperative analgesia in patients undergoing intertrochanteric hip fracture repair [35]. Even if not used as the primary anesthetic, a lumbar plexus block as an adjunct to general anesthesia compared to general anesthesia alone for hip fracture repair has been shown to improve intraoperative hemodynamics in addition to facilitating lower postoperative pain scores (1, 3, and 6 h), faster recovery times, and the decreased incidence of adverse events [36]. Although the lumbar plexus block is a great block, due to its level of difficulty and higher complication risks, its utilization has significantly dropped due to the availability of safer alternatives such as the FICB and FNB. 

### 3.6. Erector Spinae Plane (ESP) Block and Quadratus Lumborum (QLB)

Erector spinae plane (ESP) block targets the dorsal roots of the spinal nerves as they travel in the fascial plane between the transverse processes and the erector spinae muscle. The adequate volume of local anesthetic is approximately 30 mL. ESP blocks have been reported to be used with variable degrees of effectiveness for analgesia after hip surgery in general and specifically in the setting of hip fracture surgery [37]. One report described the use of a large volume of local anesthetic mixture (40 mL) to produce surgical anesthesia in 15 patients without the need to convert to general anesthesia or add more local infiltration by the surgeon [38] MRI examination of the lumbar spine after ESP block revealed a significant contrast spread between the T12 and L5 transverse process and erector spinae muscle. Contrast material spread to the paravertebral, foraminal, and partially epidural area/spaces and in the areas where the lumbar nerves enter the psoas muscle which may hint at the mechanism of analgesia of the block [38]. The lumbar ESP block has shown similar analgesia to the infra-inguinal fascial iliaca block after elective hip arthroplasty with the potential for motor sparing of the quadriceps muscle [39]. A recent meta-analysis concluded that the use of the lumbar ESP block resulted in reduced opioid consumption in the first 24 h after surgery with significantly lower pain scores in the first 8 h after surgery [40].

The quadratus lumborum (QL) block is another relatively novel fascial plane block that aims to deposit local anesthetic in different locations relative to the muscle (anterior, lateral, or posterior) under the thoracolumbar fascia where the spinal nerves and their branches will travel. Research investigating the efficacy of the QL blocks in hip fracture surgery is still evolving. Different approaches to the QL blocks using approximately 30 mL of local anesthesia have shown reduced pain scores, reduced opioid consumption, and improved patient satisfaction when used for analgesia after hip surgery [41]. One study compared lumbar ESP and QL blocks to control groups in hip and proximal femoral surgery. This study showed improved analgesic quality in patients receiving ESPBs and QLBs [42]. When the QL block was compared to the SIFI block, the latter was found to result in prolonged analgesia and less pain during positioning for spinal anesthesia [43]. However, more research is needed to understand the benefits of ESP and QL blocks, especially in the context of more powerful blocks such as SIFI, the FNB, and PENG.

### 3.7. Sciatic Nerve Block (SNB)

The hip capsule is commonly split into the anterior and posterior capsule for the purposes of exploring its nociceptive innervation. The sciatic nerve is the major nerve that supplies the posterior capsule of the hip with some contribution from the nerve to quadratus femoris and superior gluteal nerve [44]. Thus, without anesthetizing this target, it is very difficult to manage posterior hip capsule pain. The transgluteal approach was the original approach described by Labat et al. However, currently, the subgluteal approach to the nerve is more common and can be blocked using ultrasound guidance. The nerve is located in the subgluteal fascial plane above the quadratus femoris muscle between the greater trochanter and ischial tuberosity [45].

Studies exploring the sciatic nerve block for hip fracture in isolation are few, and none from the last 5 years were found. More commonly, the sciatic nerve is blocked in concert with other targets that innervate the anterior capsule. In a Chinese study, the authors compared inflammatory markers of patients split in two groups: one receiving combined spinal–epidural anesthesia and the other receiving combined the lumbar plexus–sciatic nerve block as the primary block. They showed marked reductions in the inflammatory markers studied in their population, which included 62 elderly patients [46]. As seen here, the sciatic nerve was an adjunctive portion of the total regional anesthetic. Another study showed the effect of combining lumbar plexus and sciatic nerve blocks for hip fracture surgery by comparing them to spinal anesthesia, wherein they showed that a combined lumbar plexus–sciatic nerve block was equivalent to spinal anesthesia in terms of adequate anesthesia for surgery. Their study population showed a bias toward using the regional block as the primary block for sicker patients, which is their institutional norm, and providing spinal anesthesia for those deemed healthier [47].

Like the LFCN block, the sciatic nerve block is not useful as a sole anesthetic for hip fracture surgery but can be part of a holistic approach to the hip, especially when coverage to the posterior capsule is required.

### 3.8. Superior Gluteal Nerve (SGN) and Nerve to Quadratus Femoris Muscle

The predominant approach to pain management for hip fractures is anterior capsule coverage; however, some patients still experience significant residual posterior hip pain. The superior gluteal nerve (SGN) and the nerve to the quadratus femoris muscle are nerves that supply the posterior capsule of the hip [6,48]. The superior gluteal nerve emerges cranially to the piriformis muscle and deep to the gluteus maximus muscle and craniodorsal to the triceps coxae [48]. The nerve runs along with the superior gluteal artery and then divides into two branches covering the gluteus medius and gluteus minimus muscle, thus supplying innervation for the posterior capsule of the hip. The nerve to the quadratus femoris muscle also provides sensory innervation to the posteroinferior capsule. It arises from the upper part of the sciatic nerve.

**Figure 7:**
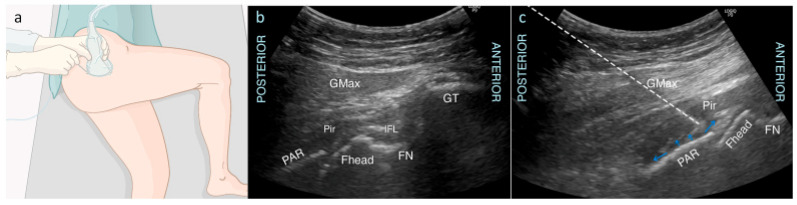
(**a**) Position of the patient during the posterior pericapsular deep-gluteal block. The ultrasound probe and needle are in line with the axis of the femur, with in-plane needle puncture from the posterior aspect. (**b**) Corresponding ultrasound image showing the gluteal muscles covering the bony landmarks: Fhead, femoral head; FN, femoral neck; GMax, gluteus maximus; GT, greater trochanter; IFL, ischiofemoral ligament as part of the posterior hip capsule; PAR, posterior acetabular rim; Pir, piriformis muscle. (**c**) Ultrasound image of the needle trajectory. Dashed line: needle trajectory; Blue arrows: spread of local anesthetic over the posterior acetabular rim and posterior hip capsule, deep to the piriformis muscle. Reproduced with permission from Vermeylen K et al., BJA Open; published by Elsevier, 2023 [48].

Vermeylen et al. from Turnhout, Belgium, developed a new ultrasound-guided technique to block the nerves to the posterior capsule called the posterior pericapsular deep-gluteal (PPD) block [48]. The block is performed with the patient positioned in the lateral decubitus position with both their knee and hip flexed at 90 degrees (Figure 7a). A low-frequency curved probe is placed on the greater trochanter and aligned parallel with the long femoral axis. The probe is then moved slightly dorsal to visualize the bony landmarks: the greater trochanter, the femoral neck and head, and the posterior acetabular rim (Figure 7b) [48]. Superficial to the bony landmarks, the piriformis muscle is identified, deep to the gluteus maximus muscle. The needle is introduced in-plane from lateral to medial until the tip contacts the posterior acetabular rim near the attachment of the ischiofemoral ligament. After negative aspiration, 20 mL of local anesthetic is injected with fluid spread over the posterior acetabular rim, the posterior hip capsule, and under the piriformis muscle (Figure 7c) [48]. With this volume, the superior gluteal nerve and the nerve to the quadratus femoris muscle is expected to be covered.

Like LFCN and PENG blocks, the SGN and nerve to the quadratus femoris muscle blocks are not useful as a sole anesthetic for hip fracture surgery but can be part of a comprehensive approach to pain management.

## 4. Surgeon’s Perspective on Nerve Blocks for Hip Fracture

While ‘hip fractures’ are often grouped together, when considering the treatment options and optimal management, there are many different pathways.

Extracapsular hip fractures, including those in the intertrochanteric region and basicervical neck fractures, are treated with reduction and fixation. However, within this category, there are several implant options to choose from. Based on the implant (and difficulty of reduction), a patient may have only a small lateral approach below the vastus ridge, several smaller incisions spread from proximal to the hip joint all the way to the distal femur, or a combination of smaller and larger incisions.

Intracapsular hip fractures can be treated with open reduction and internal fixation (in young patients), which could have one or multiple approaches, including anterior and lateral. They can also be treated with fixation in situ, which would be performed from the lateral femur below the vastus ridge. Finally, they can be treated with an arthroplasty procedure—either a hemiarthroplasty or total hip arthroplasty. These procedures can be performed through many different approaches including posterior, lateral, anterolateral, or direct anterior.

In addition to knowing what type of fracture a patient has, an essential part of assessing a hip fracture patient is to determine if there are other acute illness that led to the fall. For example, illness such as anemia, heart failure, stroke, arrhythmias, and/or a myocardial infarction will need to be treated prior to a patient undergoing surgery. A preoperative assessment involving an anesthesia and medicine team is crucial for optimal outcomes. With the variability in treatment, including the surgical approach and the invasiveness of procedure, the ultimate goal is to have patients bear weight straight after surgery. Thus, it is important to have an effective postoperative analgesia which will encourage early mobilization and rehabilitation by the physical therapist. Overall, it is critical for the surgical and anesthesia team to have excellent communication to optimize care for patients.

## 5. Conclusions

Hip fracture is a painful injury that significantly limits mobility, thus increasing morbidity, especially in the elderly population. Early surgical treatment and the use of peripheral nerve blocks have been shown to reduce morbidity and mortality. Peripheral nerve blocks such FICB, FNB, LFCN, and PENG blocks are currently the most used blocks for hip fractures (Table 1). However, other blocks like ESP, QLB, sciatic nerve, and lumbar plexus blocks can be beneficial in certain hip fractures and surgical procedures such as hip arthroplasty or hip hemiarthroplasty. In addition to traditional blocks, we may now have a new block that anesthetizes nerves to the posterior capsule, superior gluteal nerve (SGN), and the nerve to the quadratus femoris muscle called the posterior pericapsular deep-gluteal (PPD) block. With all these peripheral nerve blocks, complete analgesia of the hip is possible, thus optimizing perioperative pain management.

## Figures and Tables

**Table 1:** Peripheral nerve blocks with their advantages and disadvantages.

Peripheral Nerve Block	Advantages	Disadvantages
* Fascia Iliaca (FICB)	Blocks LFCN and FN reliably. Blocks obturator nerve more reliably with higher volume of injection. Effective analgesia, supported by several RCTs. Low risk of intravascular injection. Good for all approaches to hip surgery.	High risk of quadricep weakness due to spread to femoral nerve, especially with infra-inguinal approach. Requires larger volume (30–40 mL).
* Femoral Nerve (FNB)	Effective coverage of anterior hip joint. Effective analgesia, supported by several RCTs. Superficial and simple to perform. Moderate risk of intravascular injection.	High risk of quadricep weakness. High risk of nerve injury due to proximity to nerve on injection. May spare LFCN depending on volume.
* Lateral Femoral Cutaneous Nerve (LFCN)	Low risk of nerve injury. Low risk of intravascular injection. Good for lateral or posterior lateral approach to hip.	Need to block FN separately.
* Pericapsular Nerve Group (PENG)	Low risk of quadricep weakness. Low risk of nerve injury as it is a fascia plane block. Effective analgesia, supported by some RCTs.	Spares LFCN and femoral nerves, thus necessitating separate blocking.
Lumbar Plexus Nerve (LP)	Blocks femoral, LFCN, and obturator nerves reliably. Good for all approaches to hip surgery.	High risk of quadricep weakness. High risk of nerve injury if performed without nerve stimulator or ultrasound. Risk of bilateral and epidural spread. Higher risk of local toxicity due to absorption. Deep block; use with care in anticoagulated patients (increase peritoneum hematoma).
Quadratus Lumborum (QL)	Low risk of quadricep weakness if performed appropriately using both ultrasound and nerve stimulation.	Variable degree of evidence regarding efficacy. Requires larger volume (≅30 mL). Deep block (posterior/anterior); use with care in anticoagulated patients.
Erector Spinae Plane (ESP)	Low risk of quadricep weakness.	Variable degree of evidence regarding efficacy. Requires larger volume (≅30–40 mL).
Sciatic Nerve (SNB)	No quadricep weakness. Blocks posterior capsule of hip.	Only blocks sciatic nerve. Will need to add other blocks, i.e., FICB, FN, or LFCN, for adequate coverage of hip joint. May be uncomfortable for patients; requires premedication due to positioning.
Superior Gluteal Nerve Nerve to Quadratus Femoris Muscle	Blocks posteroinferior capsule of hip.	Not useful as sole anesthetic. Will need to block with FICB and PENG.

* Commonly used for hip fracture surgery.
